# Uptake of antepartum care services in a matrilineal-matrilocal society: a study of Garo indigenous women in Bangladesh

**DOI:** 10.1186/s12884-023-05404-z

**Published:** 2023-01-28

**Authors:** Suban Kumar Chowdhury

**Affiliations:** Department of International Relations, University of Rajshahi, Rajshahi, Bangladesh

**Keywords:** Indigenous women, Matrilineal-matrilocal, Antepartum care, External environment, predisposing, Enabling, perceived needs

## Abstract

**Background:**

The indigenous Garo is a close-knit matrilineal-matrilocal community. This community's expectant mothers receive less antepartum biomedical care, making them prone to maternal mortality. This study developed a conceptual framework to explore how the external environment, personal predispositions, enabling components and perceived antepartum care needs influence and generate a gap in antepartum biomedical care uptake.

**Methods:**

The author used qualitative data from the study area. The data were collected through conducting 24 semi-structured interviews with purposively selected Garo women. After transcribing the data, the author generated the themes, grouped them into two broader domains, and analyzed them using the grounded theory approach.

**Results:**

The emergent themes suggest adding the external environment (i.e., healthcare facilities' availability and services and culturally relevant healthcare services) to Anderson's behavioral model to understand indigenous women's antepartum care uptake disparity. Antepartum care uptake disparities arise when Andersen's behavioral model's other three drivers-personal predisposition, enabling components, and needs components-interact with the external environment. The interplay between enabling resources and the external environment is the conduit by which their predispositions and perceived needs are shaped and, thus, generate a disparity in antepartum care uptake. The data demonstrate that enabling resources include gendered power dynamics in families, home composition and income, men's spousal role, community practices of maternal health, and mother groups' and husbands' knowledge. Birth order, past treatment, late pregnancy, and healthcare knowledge are predispositions. According to data, social support, home-based care, mental health well-being, cultural norms and rituals, doctors' friendliness, affordable care, and transportation costs are perceived needs.

**Conclusions:**

Garo family members (mothers/in-laws and male husbands) should be included in health intervention initiatives to address the problem with effective health education, highlighting the advantages of biomedical antepartum care. Health policymakers should ensure the availability of nearby and culturally appropriate pregnancy care services.

**Supplementary Information:**

The online version contains supplementary material available at 10.1186/s12884-023-05404-z.

## Background

The uptake of biomedical antepartum care is crucial for optimal health during pregnancy [1]. It helps prevent and diagnose maternal morbidity and other health complications when pregnant and serves as a counseling tool to help women and their families better understand pregnancy-related care, thereby preventing maternal mortality [2–4]. Despite its importance, the utilization of biomedical antepartum care services is minimal among mothers in need [5]. This tendency is typical among socioeconomically disadvantaged mothers, who disproportionately have poor access to biomedical care [6]. While exploring the issues behind lower access to and uptake of biomedical antepartum care services among this group of mothers, an earlier study contends that it is primarily because the uptake of biomedical antepartum care is a complex behavioral issue [7]. Available studies are divided into four groups concerning the reasons that made it a complex behavioral issue. While the first group of studies found the influence of factors such as service availability, quality, cost, society's structure, health perceptions and behaviors, and women's characteristics in making the uptake of antepartum biomedical care a complex behavioral issue [8–10], the second group of scholars identified drivers includes household economic status, gender issues (i.e., unfriendly household gender relations, gender dynamics in the household, access to and control over households' financial resources, lack of decision-making autonomy, and lack of male spouses' involvement), mothers' educational level, previous care experience, and geographical proximity to healthcare centers from the residence as most influential [11–24]. In contrast, the third group of studies found that mothers' age, birth order, family size, perception of the cultural appropriateness of available care services, cultural understanding of maternal health, and cultural awareness of pregnancy health all affect antepartum care uptake by influencing women's care-seeking behavior [25–34]. Compared to these three groups of studies, the others not only addressed factors such as ethnicity, color, and socioeconomic status as responsible for making women's uptake of antepartum care-seeking as a complex behavioral issue but also purportedly claimed that due to the impact of these factors there exist a disparity in the access to and use of biomedical antepartum care in developed and developing countries [35–38]. Another study looked into the disparity in antepartum care service use between indigenous women and their non-indigenous counterparts in the context of indigenous mothers living in Guatemala and found that indigenous women have less access to and use of biomedical antepartum care services than their non-indigenous counterparts [39].

Similar disparities between indigenous and non-indigenous women's access to and utilization of prenatal care exist in Bangladesh [40]. Indigenous women exhibit a pattern of slower-than-average maternal mortality reduction. To increase indigenous women's access to antepartum care services, the government of Bangladesh launched a community-based health intervention program under the "Framework for Tribal Peoples Plan," however, the benefits are unevenly distributed across various indigenous communities living in different parts of the country [7, 41]. Consequently, indigenous mothers have lower access to antepartum care services [42]. According to studies, for instance, the proportion of indigenous moms who accessed antepartum care services for their most recent pregnancy is approximately 6.4 percent, significantly lower than the national average of 43 percent [43]. As a result, compared to non-indigenous women, the health outcomes for indigenous women are staggeringly poor [44]. Based on this disparity, prior research on how indigenous women in Bangladesh use maternal healthcare facilities appears to be more concentrated on indigenous women living in the country's hilly regions, who are regarded as the most underprivileged ethnic group in Bangladesh [7]. However, the uptake of the antepartum biomedical care services is a complex behavioral phenomenon that varies across socio-structural contexts, places, and widespread social practices prevalent among different indigenous group [45]. Thus, there is a critical need for further research considering the unique family structures and socio-cultural contexts of various underprivileged indigenous women groups residing in Bangladesh's lowlands. To fill this research gap, the current study focused on the case of the Garo indigenous women lived in the lowlands of Mymensingh, Netrakona, Gazipur, Sherpur, and Tangail [46, 47].

Compared to other indigenous groups of this country, the Garo belongs to a matrilineal-matrilocal society [47] and represents one of the few close-knit indigenous communities in the world [48]. Being significantly impacted by neighborhood culture and practices, the Garo indigenous population maintains different sociocultural traditions different from other indigenous communities and mainstream Bangladeshis [49]. Due to their matrilineal social structure, Garo women have more prestige and freedom in the home and community [50]. In this community, women can choose their male companions, and men must move into their wives' dwellings. It refers to the concept of 'matrilocal' in Garo cultural tradition [51]. In Bangladesh, where patriarchy is dominant, the Garo indigenous group offers an ideal example of a 'female-centered' household and a more 'gender-equal society' [50], which adds an extra dimension to this research. Garo indigenous moms' disadvantaged socioeconomic status, in terms of education, employment, healthcare facilities, nutrition and food security, compared to other indigenous communities in Bangladesh was another motivation for taking their case [50, 52, 53]. The rationality of the current study lies to the comparative vulnerability of the Garo indigenous women to health complication when pregnant and their less proportional access to biomedical care services [54].

The fundamental objective of the current study was to understand the antepartum care utilization pattern among the Garo indigenous women. Although a previous study found that antepartum care-seeking is less proportionate among rural Garo moms compared to urban Garo women, other indigenous women, and non-indigenous women [55], similar to a substantial number of other studies focused on maternal health care-seeking behavior [39, 40, 56, 57], it considered the indigenous women as a homogenous group. The author argues that it is insufficient to investigate the pattern of antepartum biomedical care uptake among women by considering them as a homogenous group and, thus, approached the Garo indigenous mothers as a heterogeneous group to understand one particular issue by listening to their less-heard voices. That is, how the external environment, human predispositions, enabling variables, and perceived antepartum care needs affect biomedical uptake and generate disparity. It is meant to highlight the need to discard a homogenized epistemic view about indigenous women. It may also help explain the Garo women's heterogeneity in antepartum care uptake, despite being a close-knit indigenous community.

Five sections make up the article. As discussed in the second section, Anderson's behavioral model helped build this study's conceptual framework. The third section discusses the research methodology used for this study. The following section presents the results under two sections: (1) a description of the women and disparity in their antepartum care uptakes patterns, and (2) components that explain these disparities. The fifth section, titled 'discussions and conclusion,' analyzed the results bringing previous research findings and drew a conclusion and policy options.

### Conceptual framework

The behavioral model, developed in 1968 by Ronald M. Andersen, a US medical sociologist and expert on healthcare [8], is the most frequently suggested framework for understanding healthcare service uptake [58]. It has been used to guide the examination of predictors associated with the utilization of healthcare services [56] According to this model, the uptake of healthcare services depends on three fundamental issues [8]. These are: (1) predisposing characteristics (e.g., age, family size, educational level, birth order, and health belief), (2) enabling characteristics, including income, availability of health care centers and services, the quality of the care services, and (3) need characteristics (i.e., nature of the illness, perceived understanding of health status, the perceived need for health care, and expected satisfaction from the received services). Whereas the predisposing factors reflect that different families have different patterns in using healthcare services, enabling variables highlight that even if a family is interested in utilizing health services, they must have the means (i.e., income, access, healthcare service facilities' availability) to obtain them [59]. However, the behavioral model of the uptake of healthcare services identified the perceived needs as the most influential driver of healthcare service use [8]. When it comes to the need factors related to antepartum care during pregnancy, it refers to how women experience and perceive their pregnancy health condition, symptoms of illness, and the severity of any health complications [60]. Without need factors, the predisposing and enabling drivers might be less influential in the women's uptake of antepartum care [61]. The need represents the critical driver in utilizing healthcare services [62]. Women must perceive the need for health care and believe in any healthcare service, its essentiality, and health benefits during pregnancy [59]. It directly correlates with the uptake of healthcare services [59].

A growing body of studies primarily focused on systematic reviews of the different aspects of healthcare service uptakes has recently applied Andersen's behavioral model to structure their results [63–65]. To direct the investigation of the current study, the author used this behavioral model to develop a conceptual framework that best help understands how the external environment, personal predispositions, enabling components and perceived antepartum care needs influence the uptake of biomedical antepartum care services and produce disparity in such care utilization.

## Methods

### Study design and site

The current study used a qualitative research design. Applying the case study approach, the current study took the case of Garo indigenous mothers resided in the Lengura and Nazirpur unions of the Kalmakanda Upazila of the Netrakona District of Bangladesh. Most of the 525 Garo households in Kalmakanda Upazila live in the Lengura and Nazirpur unions [66, 67]. These 'haor' (wetland) unions have inadequate transportation, communication, and biomedical healthcare service facilities for pregnant mothers. In Bangladesh, this research site is hard to reach, so the near-side availability of antepartum healthcare facilities is likely low [68]. Only motorbikes from both regions are available on the deteriorated road to the Upazila Health Complex. Against this backdrop, the author found it compelling to examine Garo indigenous women's use of antepartum care services in the Lengura and Nazirpur unions.

### Participants, selection technique and sample size

Garo women who were pregnant at the time of the data collection and who gave birth in 2020 participated in this study. Purposive sampling, specifically exponential non-discriminative snowball sampling [69] was employed to select participants. The researcher used this sampling method because it helped to expand the sample size until the data saturation by initially recruiting wave one subject who, in turn, helped to recruit wave two subjects; and thus, contributed to increasing the sample wave by wave-like a snowball growing in size as it rolls down a hill [70]. Another reason was to find the participants quickly. After interviewing 24 women in February and March 2021, the researcher noticed data saturation. So, the study sample size was limited to 24 women. Pregnancy status, social position, education, employment status, and age were considered when selecting participants. Among the participants, thirteen women were pregnant at the interview, while the other eleven had given birth in 2020. Their ages ranged from 16 to 35, with most in their late 20 s. Twelve of the twenty-four participants came from low-income homes, nine from middle-income homes, and three from high-income homes. The participants were put into three groups based on their total monthly income and the BBS data on the average monthly income per household, which is 15,988 BDT per month.

### Data collection tools and procedures

Data was collected through semi-structured interviews. The primary reason for applying such a data collection instrument was to facilitate verbal exchange when one person (interviewer) asks questions to elicit information from another [71]. Open-ended questions and one-on-one in-depth interview techniques were used to conduct the semi-structured interviews [72] which helped collect the participants' narrative descriptions. The open-ended interview questionnaires (see Appendix 1) covered the mothers' daily experiences, their perceptions of their needs to access and use healthcare facilities for maternal health morbidities, their geographic access to healthcare facilities, their prior knowledge of biomedical antepartum care, their prior pregnancy experiences, and their decision-making autonomy. The interview questions also cover cultural perspectives and attitudes about biomedical healthcare services. The interview questionnaires were written in English, translated into Mandi (the Garo native language), and back into English to check for consistency with language experts. A pre-test on nine mothers who were omitted as the study participants were conducted with trained personnel in January 2021 to verify the consistency and accuracy of the data collection tool. The final data were collected from the research location between February and March 2021 by conversationally unfolding the interview protocols to allow participants to explore relevant themes [71]. Due to the gender and cultural sensitivity of the participants and the research topic, four female Garo research assistants who were experienced in qualitative data collection and fluent in Mandi were recruited to help collect data. The research assistants' presence during interviews made participants feel comfortable and helped the researcher build trust with them. They help the researcher share the purpose of this study with participants, develop a strong understanding, faith, and effective communication with them via equitable sharing and objectivity throughout the data-collection process, store the data in an audio-recorded format, and take notes of critical issues during interviews. Interviews were flexible, keeping study objectives in mind. Each of the Interviews lasted 45–60 min. The respondents' willingness to participate in the interview was prioritized.

### Data analysis

The researcher used narrative inquiry as the analytical tool for analyzing the data. This analytical tool enabled the researcher to comprehend the experiences and obstacles that influence the Garo indigenous women's uptake of antepartum care. As part of the narrative inquiry, the audio-recorded data were transcribed and translated into English and examined whether the transcripts matched the field notes. The coding process followed it. Then, the identified codes were separated based on their similarity, incongruity, and relevance to developing emerging themes. In the next step, drawing on the objective of this study, the emerging themes were categorized and adjusted by either merging or forming new categories considering the underlying sameness and differences in the collected data. Finally, an attempt was made to constitute the narratives to analyze and explain the themes to understand the uptake of antepartum care services among the Garo indigenous women following Anderson's health care behavioral model [8].

## Results

**Fig. 1:**
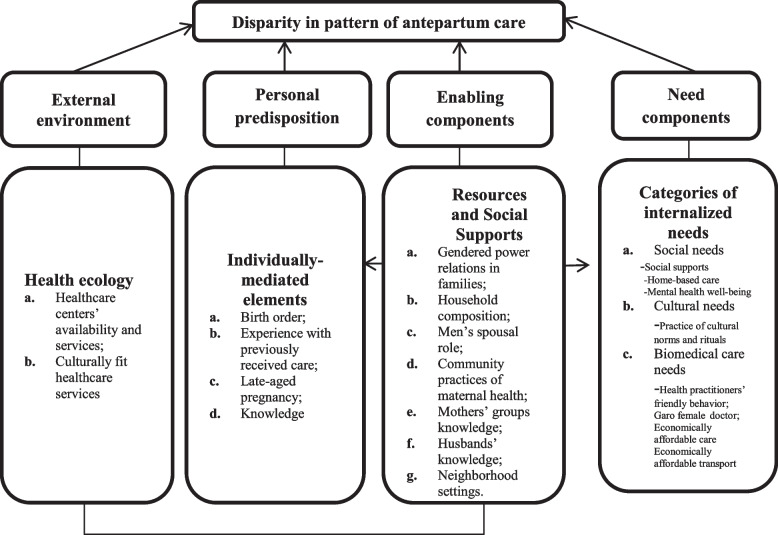
Four level of factors and emergent themes from in-dept interviews concerning the uptake of antepartum care among the Garo indigenous women

The findings are divided into two sections (see Fig. 1): (1) a description of the women and the disparity in their antepartum care patterns, and (2) components that explain these disparities. The result suggests the inclusion of an additional driver in the context of Garo indigenous women, namely the external environment, without disrupting Anderson's original model [8] (see Fig. 1). The following sections presented the results in verbatim quotations that the author has translated into English.

### The disparity in the trend of antepartum care services uptake

Most pregnant women reported feeling healthy throughout their pregnancies. However, the women could be differentiated into three groups based on their descriptions of how they used antepartum care services. In the first group, eleven Lengura Union residents with different family income levels stated they had little desire to visit the hospital when they experienced antepartum health illnesses. They relied on their mothers, in-laws, and male partners for specific antepartum health complications such as fever, urinary infection, weakness, swollen legs/body, vomiting, breathing problems, uncontrolled bleeding, and dizziness to provide in-home care. The most common types of home-based care, according to this group of women, are drinking pani pora (blessed water), consulting older women and neighbors, obtaining enough rest and spending quality time with family members, and following certain dietary taboos (i.e., not eating by sitting in the corridor, not eating certain foods).

The second group consisted of nine Nazirpur Union women who delayed seeking antepartum biomedical care when they became ill. Among this group of women, five were from low-income homes, three were from middle-income households, and one was from a high-income household. They listed all the illnesses reported by the first group of women and said home-based care was the first step to finding remedies. This women's group discussed that they only sought medical care for pregnancy health complications that did not improve with home remedies or for illnesses instantly viewed as severe, such as the abnormal fetus position in the womb and prolonged labor pain.

In contrast to the first two groups, the third group addressed the uptake of antepartum biomedical care as an essential preventive intervention for a safe pregnancy. All four mothers of this group were in their late-aged pregnancies. Three of these women lived in Nazirpur Union and hailed from wealthy families, while one lived in Lengura Union and had a similar background. This women's group uses weekly weight and blood pressure checks, three-time ultra-sonograms, and blood testing as their antepartum biomedical care.

### Factors influencing women's antepartum care uptake

#### Women’s antepartum care needs and predisposing components

As evident from the data, one group of women needed biomedical treatment for a safer pregnancy, whereas the other needed traditional remedies. According to the analysis, women's antepartum care needs differ owing to their understanding of antepartum health complications. The number of birth orders and types of antepartum care received in earlier pregnancies, pregnancy at a late age, and a knowledge gap on biomedical care advantages are significant determinants in understanding pregnancy health risks and antepartum care needs. It is how eleven women in the Lengura Union, from various socioeconomic backgrounds, had had previous pregnancies and received care at home in their previous pregnancies; understanding pregnancy as something normal and natural in a woman's life highlighted several needs to get home-based remedies for health complications when pregnant. These needs are classified here into two broad categories: first, the social needs, and second, the cultural needs. The sub-theme of cultural needs is the practices of cultural norms and rituals. Two subthemes emerged from social needs: mental health well-being and social support. Regarding social needs, the first group of women uniformly stated that family (mothers, in-laws, and male spouses) and neighboring women's supports are essential. As the preceding section mentions, it helps with natural home remedies for pregnancy difficulties; therefore, no pregnancy disease should be taken seriously. As one of the women from this group reported:

Jak jachak dalya (swelling of the body), Wakkalna ha'sika (vomiting), Matha Betha (Dizziness), Hapani (shortness of breath), Durbolota (weakness), baccha nosto hoye jawa (miscarriage), Bikma Chikki Sadika (abdominal discomfort) and unexpected Mashik (menstruation) are no major health issues. These are typical pregnancy symptoms and consequences. My first and second pregnancies had these symptoms. From my experience, your mother, in-law, and husband should be consulted. She adds that advice from neighbors with previous pregnancies speeds up healing. With these supports, a pregnant woman shouldn't worry. (P1, 14 February 2021).

Furthermore, their discussion suggests that mental wellness is essential for a safe pregnancy. Also, mental happiness during pregnancy helps improve the fetus's health. This group of women often cites familial support as crucial to mental health well-being. As one of the participants of this group puts it:

Minds need to be refreshed during pregnancy. A mentally healthy woman won't get sick during pregnancy. The mother's nari (umbilical cord) is linked to the baby. If maa (mother) is happy, so is the baby. She needs support from her mother, husband, neighbors’ women, and relatives to be mentally happy when pregnant (P4, middle-class household, age-25).

The same group of women emphasizes cultural needs while discussing spiritual and non-spiritual threat-free pregnancy. They also acknowledged that cultural rituals are essential to meeting this need. These women feel that safer pregnancy means no non-spiritual symptoms or spiritual threats. Cultural norms and rituals are the only way to ensure a healthy pregnancy without spiritual or non-spiritual threats. They related the signs and dangers of spiritual and non-spiritual threats to physical health during pregnancy. Dizziness, vomiting, body swelling, and weakness linked to internal changes during pregnancy are non-spiritual, meaning they come and usually go if one follows cultural norms and traditions. The spiritual threat causes abdominal pain, shortness of breath, miscarriage, cramping, and bleeding during pregnancy. It risks a miscarriage or death for pregnant women. As one of the women in the group put it:

Our community values some niyom (rules). Safe pregnancy requires these niyoms. These niyoms (rules) are essential to follow to avoid symptoms like jak jachak dalya (body swelling), Wakkalna ha'sika (vomiting), matha betha (daze), Hapani (shortness of breath), Bikma Chikki Sadika (abdominal discomfort), and durbolota (weakness), as well as shoitan's (evil force's) attacks that induce miscarriage (P6, low-class household, age-28).

When discussing the importance of cultural norms and traditions in preventing spiritual threats and non-spiritual illnesses, participant P1 says:

To evade Shoitan's attacks, Niyoms (rules) must be obeyed. My neighbor lost her baby because she didn't follow the safe pregnancy Niyoms. Shoitan stole her baby in the seventh month (miscarriage). Thus, I realize the effects. The Niyoms will help me avoid the Shoitan. She says, "yeah, I understand that vomiting, dizziness, and weakness do not arise due to Shoitan's attack, and they are not severe illnesses, but rather the regular indicators." All pregnant women have matritto (motherhood) symptoms. These result from body changes. During my pregnancy, Niyoms helped me get rid of them. In this case, daktars (doctors) are unnecessary. She says, "It's not just me expressing it." My female neighbors had the same experience. However, daktars' concerns were ignored. So why should I? (P1, low-class family, age-27).

The women in the first group thus justify their uptake of home-based remedies based on the aforementioned socio-cultural needs that emerged from their prior pregnancies and experiences with care.

In the second group of women with skilled antepartum care needs, personal predispositions such as late-aged pregnancy and an understanding of the advantages of biomedical care appeared as two interesting factors in justification of the uptake of hospital-level antepartum care services. There are thirteen women in this group (twelve from Nazirpur Union who were from different household income statuses and one from the Lengura Union with privileged household income status). Four of the women in this group, three from the Nazirpur Union and one from the Lengura Union, who became pregnant after turning 35, addressed their pregnancy health problems more candidly and revealed more awareness of their health needs:

I am 35 years old and six months pregnant. I am very aware of my health needs because I know how risky being pregnant at an elderly age is. Yes, I agree that seeking care from a qualified doctor is essential if you want to be healthy while pregnant. Additionally, it promotes the fetus's quick and healthy growth (Q9, middle-class household, age-35).

Like late-aged pregnant women, the other group members were better at recognizing antepartum health complications' causes and consequences. They could see the potential to improve pregnancy health and well-being whether or not they employed their community's resources. Most women in this group with high household incomes said that going to a hospital far from their villages harms their pregnant health due to the flawed road communication system. Many women also complain about doctors' improper behavior and the lack of Garo female doctors. They linked these issues to their mental health and reported:

I know pregnancy needs medical care. Being pregnant, distance and the long trip could endanger my pregnancy. Another thing: going to the doctor seems like a mental burden right now, which is unsafe. These mental stressors include the hospital's unpleasant, non-Garo male doctors. So, only after home cures failed, I went to the hospital (Q5, high-class household, age-24).

In addition, low-income women delayed treatment because of the high cost of hospital-level care and costly transportation to the hospital:

My spouse and I are poor, so monthly hospital visits are difficult. Doctors and medicines are too expensive for me. No Garo female doctors exist, and the male doctors' bad behavior endangers pregnant women's emotional well-being. With this in mind, I mainly go to the doctor when home remedies fail (Q1, Low-class household, Age 19).

Regardless of income, most of this group's women favored biomedical care following home cures. It suggests that the Garo indigenous women's needs-friendly health practitioners, care from female health practitioners in their community and inexpensive transportation and care facilities-were more critical in determining their medicinal health care uptake. This group of women's unmet antepartum care needs also explained why they did not use the Upazila hospital's competent and preventative healthcare services.

### Enabling components and external environment

One key theme emerged from the participants' stories: (1) 'gender-friendly relations' in the home between husband and wife, mothers and daughters, or mothers in-laws and daughters in-laws.

Findings demonstrate that matrilineal-matrilocal societies value elderly adults as representatives of gendered power relations. Moms and mothers-in-law in extended homes make the most critical antepartum healthcare-related decisions, according to the study. However, the household decision-making process helps meet pregnant mothers' related care needs. Cultural acknowledgment of family bonds leads to participatory antepartum healthcare-related decision-making, as evidenced by the women's discussions. In such a process, the daughters/in-laws can also participate. They are free to consult older adults regarding their pregnancy care-related matters. According to one woman:

As family head, my mother makes all health decisions. Since he's a man, my husband also participates. But they only make decisions after consulting me (P2, middle-class household, age-30).

Women's narratives showed a pattern in collaborative decision-making structures for antepartum care uptake. Older women and male spouses' opinions and decisions concerning antepartum care are highly valued. The universal respect for older female adults and male spouses and the customary societal practice of recognizing their choices contribute to a general predisposition to follow what husbands and mothers/in-laws say after a joint discussion on antepartum care matters. Thus, mothers' and male spouses' perspectives and neighborhood antepartum care practices positively affect women's care needs throughout pregnancy and their willingness to use care services. In this regard, two groups emerged from participants' narratives. First, nine Nazirpur Union ladies said their mothers/in-laws advised them to seek biomedical care only when home-based remedies failed. Male husbands and mother groups were concerned about the unavailability of close health centers and the distance to the Upazila hospital, which these women frequently stated as grounds for delaying biomedical antepartum care uptake. As participant Q4 states:

Yes, she (mother) always advised me about Daktari care (doctors' care); she explained to me that other women from my area also got similar treatment during pregnancy; therefore, I should follow the practice (Q4, middle-class household, age-28).

This group of women also reported that their male spouses and mothers/in-laws always talked about the cultural and social value of the care offered by Garo female health practitioners in the same community. As evidenced in these women's discussions, their male partners and mothers/in-laws convinced them that exposing a woman's body to any male doctor for health checkups and discussing pregnant health issues with a doctor is against womanhood because they are womanly issues. They often mentioned that their husbands and mothers/in-laws had advised them that talking with male health practitioners about such matters was against societal practice. Their community did not use non-Garo female doctors for antepartum treatment. These women's narratives also emphasized their husbands', mothers'/in-laws', and neighborhoods' views of Garo female health practitioners' efficacy and fellow emotions. Thus, gender and ethnic socialization at the family level and the perceived fellow feeling and healthcare service delivery efficacy of the Garo female doctors interact with the health ecology of their residences and affect predisposing factors that affect antepartum care uptake:

My mother feels pregnancy is a Meyeli (womanly) issue; hence a Garo female Daktar (doctor) is essential. She said our whole community goes to Garo female Daktar. I should also follow this (Q6, low-class household, age-24).

However, eleven women from the Lengura Union reported that their mothers' group and male partners routinely educate them on cultural norms and customs when discussing pregnancy health issues. As one of the participants put it:

My mother warned me to avoid Shoitan's impact (evil eyes). She advised expecting mothers to prevent the Shoitan. She also asks if I agree or have other ideas. But I said no because I witnessed how successfully my neighbors' pregnancies went by following their mothers' care guidelines (P6, low-income household, age-28).

When it comes to community practices on pregnancy health care, 'home burden-sharing and taking care duties' has emerged as a predominant topic from the participants' narratives related to the first theme. Extended-family Garo women receive a great deal of support when pregnant. In joint families, they share household responsibilities with husbands and elderly adults. Thus, they have more time to rest and visit health centers to avoid pregnancy-related health complications than Garo women who live in nuclear households. According to participant Q3:

I don't worry about my husband's food. My mom said I don't have to work because I'm pregnant. My husband and mother advised me to rest (Q3, joint family, age 19).

For those women who reside in their mothers-in-homes, respondent P10 states the following:

My family responsibilities do not prevent me from obtaining pregnancy care. My mother-in-law and husband are concerned about me. They say I shouldn't perform chores since it can make me sick. (P10, middle class, extended family, husband's residence, age 26).

Even if one's spouse is a migrant worker who only visits once a month, sharing the household's work burden and taking care of a pregnant mother is so ingrained in Garo indigenous community pregnancy healthcare that it does not matter. In such a situation, extended family members never made her feel alone. They offer emotional support and uphold shared household responsibilities. Thus, migrant workers' spouses in extended families receive superior antepartum care than those in single households. According to respondent P1:

He (her husband) pulls rickshaws in Dhaka. He generally comes twice a month. I don't mind because my mother and sisters are very loving. They won't let me do anything now. They, like my husband, always advise me to rest (P1, low-class household, joint family, age-27).

Thus, the research revealed two insightful points. Garo indigenous women receive antepartum care from their families due to gender-friendly household dynamics and community practices of sharing domestic chores. Mothers' groups and male partners encourage women to seek care. Second, family support and community practices on pregnancy health care were associated with the distinction between home-treatable and hospital-level treatable pregnancy complications when pregnant.

## Discussions and conclusion

The method used in this study aids in understanding how the external environment, predisposing, enabling, and perceived needs interact with multiple levels to generate disparity in antepartum care utilization. Anderson's model [8] of healthcare use was used to build this framework, revealing two links: one between health ecology and enabling resources, and another among the individual predisposition, enabling components, and perceived need. By drawing these connections, the author explored how these four components affect antepartum care service utilization patterns. This technique goes a step further by including the external environment's effect into Anderson's behavioral model to see how disparity in antepartum care uptake among women in close-knit female-centered indigenous communities with a shared culture, such as Garo indigenous women. When these connections are revealed, the complicated ways in which antepartum care uptake variations emerge.

In contrast to a substantial number of previous studies demonstrate the less proportional uptake of antepartum care among indigenous women by portraying them as a homogenous group [39, 40, 55–57], the current study reflects the heterogeneity of the indigenous women concerning the utilization of antepartum care, and it is despite their belonging to a close-knit community setting different from other indigenous groups. The majority of women in the Lengura Union are more experienced with home-based care solely, and hence biomedical care appears to be less relevant to them, according to one main finding of this study. In contrast, another group of women, primarily from the Nazirpur Union, commonly use home-based remedies as their first line of defense, delay biomedical treatment, and underutilize their knowledge of biological antepartum care services. Similarly, women who had their pregnancy at a later age appear to be more likely to employ biomedical care services as a preventative approach to ensuring health and well-being while pregnant. Consistent with Anderson's model [8], in this study, evidence suggests that variation in these three groups of women's uptakes of antepartum care services is shaped by a combination of factors, including external environment, predisposing components, enabling resources, and perceived needs. In particular, the connection between personal predisposition, enabling resources, and perceived need appeared evident from the women's narratives in shaping their choices related to the uptake of antepartum care. Previous studies show that gendered power relations in the household [19, 22], lack of decision-making autonomy [13, 21, 24], and lack of men's involvement in their women's pregnancy health care matters [11, 12] are three significant barriers in women's uptake of antepartum care services. In contrast to these earlier studies, the current study provides an important insight. It is the mother groups' and male spouses' groups' knowledge about the benefit of the utilization of biomedical antepartum care, worries about the risk of going to the hospital due to the distance from their home, and the non-availability of nearside healthcare centers and the neighborhood setting that plays the influential role in the women's uptake of skilled antepartum care services. It is despite the fact that they enjoy gender-friendly household relations, equally allowed to participate in the decision-making related to pregnancy health matters, and the active involvement of the male spouses in taking care of their wives when pregnant. Specifically, the family members' beliefs about the cultural inappropriateness of the given biomedical care services were also the key to the Garo women's choices regarding their health care.

In addition, confirming the findings from previous studies conducted among indigenous women in other socio-structural contexts [31, 32], the results of this study suggest that the Garo indigenous women’s uptake of healthcare services was further limited by the deeply rooted belief of their mothers/in-laws and male spouses that pregnancy is a normal phenomenon in every woman’s life. Thus, similar to earlier studies [33, 34], this reinforced the principle that pregnancy health risk perceptions are embedded in the community’s cultural understanding of pregnancy health. The area-level difference of the family members’ culturally rooted health beliefs and its influence on Garo indigenous pregnant women’s antepartum care-seeking behavior is also reflected in the disparity in the related care services uptake. That is, whereas most of the women residing in the Lengura Union are found extensively using home-based care, including proper rest and practicing the cultural norms and rituals, the same community’s women living in the Nazirpur Union are utilizing biomedical care services depending on the effectiveness of home-based remedies in improving their health conditions. Although, several other studies also found the same pattern, which appears to be typical both in many developed, and developing countries and among many indigenous communities [37, 38, 60], the prevalent difference in the antepartum care needs and, thus, the uptake of related care services is an exceptionally interesting finding of the present research. Such a result is well backed by the argument that the pattern of women’s antepartum care services uptake depends on the socio-structural context and the prevalent social practices [45]. However, the difference in the utilization pattern of antepartum care owing to the incongruity in the pregnancy healthcare practices within the neighborhood settings raises interesting queries when considering the overall affordability of biomedical care services. Likewise, what was observed in other contexts [35, 40, 57], the Garo indigenous women's uptake of home-based care/biomedical care was not due to mere individual preference but, more importantly, by the overall consideration of the family members' choices that derived from the standard practices within the neighborhood networks. Therefore, health policymakers should incorporate Garo family members (i.e., mothers/in-laws and male husbands) in health intervention programs to address the issue with effective health education that explains the benefits of biomedical antepartum care. Also, health policymakers should take the initiative to ensure the availability of nearside and culturally fit pregnancy care services. It would contribute to reducing disparities in the utilization of related skilled care.

## Supplementary Information

**Additional file 1.**

## Data Availability

Data were collected as audio recordings, and then the author transcribed the audio recording. Datasets (audio recordings and transcriptions) used in the current study are available from the corresponding author upon reasonable request.

## References

[1] Maru S, Glenn L, Belfon K, Birnie L, Brahmbhatt D, Hadler M (2021). Utilization of Maternal Health Care Among Immigrant Mothers in New York City, 2016–2018. J Urban Health.

[2] Chauhan BG, Jungari S (2021). Factors affecting the utilization of maternal and child health care services in tribal dominated population states of India. Int Q Community Health Educ.

[3] Singh P, Singh KK, Singh P (2021). Maternal health care service utilization among young married women in India, 1992–2016: trends and determinants. BMC Pregnancy Childbirth.

[4] Usman M, Anand E, Siddiqui L, Unisa S (2021). Continuum of maternal health care services and its impact on child immunization in India: an application of the propensity score matching approach. J Biosoc Sci.

[5] Yadav AK, Sahni B, Jena PK (2021). Education, employment, economic status and empowerment: implications for maternal health care services utilization in India. J Public Aff.

[6] OrigliaIkhilor P, Hasenberg G, Kurth E, Asefaw F, Pehlke-Milde J, Cignacco E (2019). Communication barriers in maternity care of allophone migrants: Experiences of women, healthcare professionals, and intercultural interpreters. J Adv Nurs.

[7] Islam RM (2017). Utilization of maternal health care services among indigenous women in Bangladesh: a study on the Mru tribe. Women Health.

[8] Andersen R, Newman JF (1973). Societal and individual determinants of medical care utilization in the United States. Milbank Mem Fund Q Health Soc.

[9] Andersen R, Newman JF (2005). Societal and individual determinants of medical care utilization in the United States. Milbank Q.

[10] Kroeger A (1983). Anthropological and socio-medical health care research in developing countries. Soc Sci Med.

[11] Kululanga LI, Sundby J, Chirwa E (2011). Striving to promote male involvement in maternal health care in rural and urban settings in Malawi-a qualitative study. Reprod Health.

[12] Ladur AN, van Teijlingen E, Hundley V (2021). Male involvement in promotion of safe motherhood in low-and middle-income countries: a scoping review. Midwifery.

[13] Gautam S, Jeong HS (2019). The role of women’s autonomy and experience of intimate partner violence as a predictor of maternal healthcare service utilization in Nepal. Int J Environ Res Public Health.

[14] Almeida APSC, Nunes BP, Duro SMS, Facchini LA (2017). Socioeconomic determinants of access to health services among older adults: a systematic review. Rev Saude Publica.

[15] Srivastava D, McGuire A (2016). The determinants of access to health care and medicines in India. Appl Econ.

[16] Mezieobi SA, Ibekwe P. Contemporary family choice: Areas of Nigerian families’ change lag. Eur J Res Reflect Educ Sci. 2017;5(1):66–70.

[17] Hailemariam S, Agegnehu W, Derese M (2021). Exploring COVID-19 related factors influencing antenatal care services uptake: a qualitative study among women in a rural community in Southwest Ethiopia. J Prim Care Community Health.

[18] Zegeye B, El-Khatib Z, Ameyaw EK, Seidu AA, Ahinkorah BO, Keetile M (2021). Breaking barriers to healthcare access: a multilevel analysis of individual-and community-level factors affecting women’s access to healthcare services in Benin. Int J Environ Res Public Health.

[19] Banda PC, Odimegwu CO, Ntoimo LFC, Muchiri E (2017). Women at risk: Gender inequality and maternal health. Women Health.

[20] Rosário EVN, Gomes MC, Brito M, Costa D (2019). Determinants of maternal health care and birth outcome in the Dande Health and Demographic Surveillance System area, Angola. PLoS One.

[21] Osamor PE, Grady C (2016). Women’s autonomy in health care decision-making in developing countries: a synthesis of the literature. Int J Womens Health.

[22] Morgan R, Tetui M, MuhumuzaKananura R, Ekirapa-Kiracho E, George AS (2017). Gender dynamics affecting maternal health and health care access and use in Uganda. Health Policy Plan.

[23] Okonofua F. Maternal Mortality in Developing Countries BT - Contemporary Obstetrics and Gynecology for Developing Countries. In: Okonofua F, Balogun JA, Odunsi K, Chilaka VN, editors. Cham: Springer International Publishing; 2021. p. 13–22. Available from: 10.1007/978-3-030-75385-6_3.

[24] Dev R, Williams-Nguyen J, Adhikari SP, Dev U, Deo S, Hillan E (2021). Impact of maternal decision-making autonomy and self-reliance in accessing health care on childhood diarrhea and acute respiratory tract infections in Nepal. Public Health.

[25] Chakraborty N, Islam MA, Chowdhury RI, Bari W, Akhter HH. Determinants of the use of maternal health services in rural Bangladesh. Health Promot Int. 2003;18(4):327–37. 10.1093/heapro/dag414 14695364

[26] Yosef T, Tesfaye M (2021). Pregnancy danger signs: Knowledge and health-seeking behavior among reproductive age women in southwest Ethiopia. Women’s Health.

[27] Woldemicael G, Tenkorang EY (2010). Women’s autonomy and maternal health-seeking behavior in Ethiopia. Matern Child Health J.

[28] Guo F, Xiong H, Qi X, Takesue R, Zou Siyu BM, He Qiwei P (2021). Maternal health-seeking behavior and associated factors in the Democratic Republic of the Congo. Health Educ Behav.

[29] Blanford JI, Kumar S, Luo W, MacEachren AM (2012). It’sa long, long walk: accessibility to hospitals, maternity and integrated health centers in Niger. Int J Health Geogr.

[30] Ibrahima AB (2021). Exploring Maternal Health in Ethiopia Using Indigenous Approaches: Policy and Practice Implications. Res Soc Work Pract.

[31] Schwartz DA. Introduction to Indigenous Women and Their Pregnancies: Misunderstood, Stigmatized, and at Risk BT - Maternal Death and Pregnancy-Related Morbidity Among Indigenous Women of Mexico and Central America: An Anthropological, Epidemiological, and Biomedical Approach. In: Schwartz DA, editor. Cham: Springer International Publishing; 2018. p. 3–9. Available from: 10.1007/978-3-319-71538-4_1.

[32] Chamberlain C, McNamara B, Williams ED, Yore D, Oldenburg B, Oats J (2013). Diabetes in pregnancy among indigenous women in Australia, Canada, New Zealand and the United States: a systematic review of the evidence for screening in early pregnancy. Diabetes Metab Res Rev.

[33] Adeleye OA, Aldoory L, Parakoyi DB (2011). Using local culture and gender roles to improve male involvement in maternal health in southern Nigeria. J Health Commun.

[34] Barnes LAJ, Barclay L, McCaffery K, Aslani P (2018). Complementary medicine products used in pregnancy and lactation and an examination of the information sources accessed pertaining to maternal health literacy: a systematic review of qualitative studies. BMC Complement Altern Med.

[35] Adhikari T, Sahu D, Nair S, Saha KB, Sharma RK, Pandey A (2016). Factors associated with utilization of antenatal care services among tribal women: A study of selected States. Indian J Med Res.

[36] Leonard SA, Main EK, Scott KA, Profit J, Carmichael SL (2019). Racial and ethnic disparities in severe maternal morbidity prevalence and trends. Ann Epidemiol.

[37] Westgard CM, Rogers A, Bello G, Rivadeneyra N (2019). Health service utilization, perspectives, and health-seeking behavior for maternal and child health services in the Amazon of Peru, a mixed-methods study. Int J Equity Health.

[38] Ononokpono DN, Odimegwu CO (2014). Determinants of maternal health care utilization in Nigeria: a multilevel approach. Pan Afr Med J.

[39] Chomat AM, Solomons NW, Montenegro G, Crowley C, Bermudez OI (2014). Maternal health and health-seeking behaviors among indigenous Mam mothers from Quetzaltenango, Guatemala. Revista Panamericana de Salud Pública.

[40] Akter S, Davies K, Rich JL, Inder KJ (2020). Barriers to accessing maternal health care services in the Chittagong Hill Tracts, Bangladesh: a qualitative descriptive study of Indigenous women’s experiences. PLoS One.

[41] Bhowmik KR, Das S, Islam MA (2020). Modelling the number of antenatal care visits in Bangladesh to determine the risk factors for reduced antenatal care attendance. PLoS One.

[42] Uddin J, Pulok MH, Johnson RB, Rana J, Baker E (2019). Association between child marriage and institutional delivery care services use in Bangladesh: intersections between education and place of residence. Public Health.

[43] Akter S, Rich JL, Davies K, Inder KJ (2019). Access to maternal healthcare services among Indigenous women in the Chittagong Hill Tracts, Bangladesh: a cross-sectional study. BMJ Open.

[44] Adnan S, Dastidar R (2011). Alienation of the lands of indigenous peoples.

[45] Hyzam D, Zou M, Boah M, Saeed A, Li C, Pan S (2020). Health information and health-seeking behaviour in Yemen: perspectives of health leaders, midwives and mothers in two rural areas of Yemen. BMC Pregnancy Childbirth.

[46] Hossain S, Begum JA, Banu MLA, Rahman MF, Akhter Z (2010). Prediction of Stature From Hand Length and Breadth An Anthropometric Study on Christian Garo tribal Bangladeshi females. Bangladesh J Anatomy.

[47] Hasan M, Momtaz P, Hosen I, Das SA, Akhteruzzaman S (2015). Population genetics of 17 Y-chromosomal STRs loci in Garo and Santal tribal populations in Bangladesh. Int J Legal Med.

[48] Dey S, Resurreccion BP, Doneys P (2014). Gender and environmental struggles: voices from Adivasi Garo community in Bangladesh. Gend Place Cult.

[49] Haque MM, Mandal S, Sultana J (2015). Nutritional status and associated socioeconomic factors of 15–49years garo ethnic women residing in northern part of Bangladesh: a cross sectional observational survey. Age (Omaha).

[50] Karim MA, Kabir MM, Siddiqui MA, Laskar MSI, Saha A, Naher S (2019). Epidemiology of Imported Malaria in Netrokona District of Bangladesh 2013–2018: Analysis of Surveillance Data. Malar Res Treat.

[51] Das TK, Islam S (2005). Psycho-social dimensions of ethnicity: The situation of Garo community in Bangladesh. Asian Aff (Lond).

[52] Munro J, Parker B, McIntyre L (2014). An intersectionality analysis of gender, indigeneity, and food insecurity among ultrapoor Garo women in Bangladesh. Int J Indig Health.

[53] Tamanna S, Rana MM, Ferdoushi A, Ahmad SAI, Rahman M, Rahman A (2013). Assessment of nutritional status among adolescent Garo in Sherpur District, Bangladesh. Bangladesh J Med Sci.

[54] Navile TE, Hossain J (2019). Birth ritual, practices and gender preference for expecting child in a matrilineal indigenous community: a case of Garos living in Sainamari village, Madhupur, Bangladesh. J Asia Soc Bangladesh (Hum).

[55] Rahman SA, Kielmann T, McPake B, Normand C (2012). Healthcare-seeking behaviour among the tribal people of Bangladesh: can the current health system really meet their needs?. J Health Popul Nutr.

[56] Akter S (2022). Factors influencing health service utilization among mothers for under-five children: a cross-sectional study in Khulna district of Bangladesh. PLoS One.

[57] Akter S, Davies K, Rich JL, Inder KJ (2019). Indigenous women’s access to maternal healthcare services in lower-and middle-income countries: a systematic integrative review. Int J Public Health.

[58] Ricketts TC, Goldsmith LJ (2005). Access in health services research: the battle of the frameworks. Nurs Outlook.

[59] Fosu GB (1994). Childhood morbidity and health services utilization: cross-national comparisons of user-related factors from DHS data. Soc Sci Med.

[60] Ononokpono DN, Odimegwu CO, Imasiku E, Adedini S (2013). Contextual determinants of maternal health care service utilization in Nigeria. Women Health.

[61] Chakraborty N, Islam MA, Chowdhury RI, Bari W, Akhter HH (2003). Determinants of the use of maternal health services in rural Bangladesh. Health Promot Int.

[62] Fiedler JL (1981). A review of the literature on access and utilization of medical care with special emphasis on rural primary care. Soc Sci Med C.

[63] Roberts T, Miguel Esponda G, Krupchanka D, Shidhaye R, Patel V, Rathod S (2018). Factors associated with health service utilisation for common mental disorders: a systematic review. BMC Psychiatry.

[64] Lederle M, Tempes J, Bitzer EM (2021). Application of Andersen’s behavioural model of health services use: a scoping review with a focus on qualitative health services research. BMJ Open.

[65] Okedo-Alex IN, Akamike IC, Ezeanosike OB, Uneke CJ (2019). Determinants of antenatal care utilisation in sub-Saharan Africa: a systematic review. BMJ Open.

[66] Sharmin S (2011). Socio-Economic Situation and Land Rights of the Indigenous People in Bangladesh. OIDA Int J Sustain Dev.

[67] Rahman SMB, Uddin MB, Hussain I (2011). Anthropometric study on children of Garo and non-Garo families in Netrakona district of Bangladesh. J Bangladesh Agric Univ.

[68] Rahman AE, Perkins J, Mazumder T, Haider MR, Siddique AB, Capello C (2019). Capacities of women and men to improve maternal and newborn health: effect of a community-based intervention package in rural Bangladesh. J Glob Health.

[69] Creswell JW (2012). Collecting qualitative data. Educational Research: Planning, Conducting, and Evaluating Quantitative and Qualitative Research Fourth ed Boston. Pearson.

[70] Etikan I, Alkassim R, Abubakar S (2016). Comparision of snowball sampling and sequential sampling technique. Biom Biostat Int J.

[71] Longhurst R (2003). Semi-structured interviews and focus groups. Key Methods Geogr.

[72] Turner III DW, Hagstrom-Schmidt N. Qualitative interview design. Howdy or Hello? Technical and Professional Communication. 2022;

