# Supplementary material for: Uptake of antepartum care services in a matrilineal-matrilocal society: a study of Garo indigenous women in Bangladesh
Source: BMC Pregnancy Childbirth. 2023 Jan 28;23:75. doi: 10.1186/s12884-023-05404-z (PMC9883956; doi:10.1186/s12884-023-05404-z)
Supplement: Supplementary file 1 — Additional file 1. [file 12884_2023_5404_MOESM1_ESM.docx]

APPENDIX 1

IDI Checklist for Garo Women who were pregnant during taking the interviews and who gave birth of her child not more than 1 year before the date of taking the interview

| \| Date:  Current Age:  Maternity Status:  Age During Pregnancy:  Number of Pregnancy: \| Educational Status:  Occupation:  Living Place:  Family Type:  Family Size: \| \| --- \| --- \| |  |
| --- | --- | --- | --- |

1. Who is the earner in your family?

i. How much money did your household earn last month?

2. Describe your family's decision-making process on healthcare issues.

3. As a pregnant woman, how frequently do/did you see doctors or traditional healers for health care? If not, why not?

• Would you prefer/preferred visiting a female or male doctor/traditional healer? Why?

4. Describe your experiences with the hospitals’ healthcare services.

8. Have you encountered any health complications or illnesses during your pregnancy? Why? what are these? How does/did it occur?

9. What do/did you typically do/did when confronted with a health complication while pregnant?

i. Is/was there any specific pregnancy-related complication for which you would prefer/preferred to seek healthcare from a doctor/traditional healer?

10. What are/were you doing to maintain your health?

i. Has anyone ever taken the time to sit down with you and explain pregnancy health matters to you? Why?

• Summarize what was discussed.

11. Who is/was looking after your health in your family? How and Why?

12. What are our family's traditional and cultural attitudes towards health care throughout pregnancy?

i. Do you believe in these as well? Why?

13. If you have other pregnancy experiences-

i. How are your pregnancy experiences different from/similar to each other’s?

ii. How are your previous pregnancy experiences assisting you in next pregnancies?

14. Do you have anything further to add?

**Thank you for your time and cooperation!**
